# Draft Genome Sequence for *Frankia* sp. Strain BMG5.11, a Nitrogen-Fixing Bacterium Isolated from Elaeagnus angustifolia

**DOI:** 10.1128/MRA.00824-20

**Published:** 2020-09-10

**Authors:** Faten Ghodhbane-Gtari, Erik Swanson, Abdellatif Gueddou, Stephen Simpson, Krystalynne Morris, W. Kelley Thomas, Maher Gtari, Louis S. Tisa

**Affiliations:** a Institut National des Sciences Appliquees et de Technologie, Université Carthage, Tunis, Tunisia; b Laboratoire Microorganismes et Biomolecules Actives (LR03ES03), Faculté des Sciences de Tunis, Université Tunis El Manar, Tunis, Tunisia; c University of New Hampshire, Durham, New Hampshire, USA; University of Arizona

## Abstract

*Frankia* sp. strain BMG5.11, which was isolated from Elaeagnus angustifolia nodules, is able to infect other actinorhizal plants, including *Elaeagnaceae*, *Rhamnaceae*, *Colletieae*, *Gymnostoma*, and *Myricaceae*. Here, we report the 11.3-Mbp draft genome sequence of *Frankia* sp. strain BMG5.11, with a G+C content of 69.9% and 9,926 candidate protein-encoding genes.

## ANNOUNCEMENT

Soil-dwelling actinobacteria of the genus *Frankia* form an endophytic symbiosis with actinorhizal plants from eight angiosperm families (1, 2). This symbiosis allows actinorhizal plants to play an important ecological role as pioneer species recolonizing under harsh environmental conditions (3, 4). Molecular phylogenetic approaches have identified four major *Frankia* lineages, which also follow host plant specificity groups (5–8), and genomes from each lineage have been sequenced (9). These *Frankia* genome databases have enabled the use of “omics” approaches (10–12) and have allowed species identification of the genus (13). Of the four lineages, *Frankia* lineage 3 strains are considered to have the widest plant host range, and they also show great diversity (8) with the potential for many new species. *Frankia* sp. strain BMG5.11 was isolated, through a baiting strategy, from Elaeagnus angustifolia nodules from Tunisian soil (14). The soil came from the Gafsa region of Tunisia and was used to inoculate *E*. *angustifolia* plants. The strain was isolated on defined propionate medium (15) and was stored at −80°C. The strain was sequenced to provide more information about the diverse *Frankia* lineage 3 strains.

Sequencing of the draft genome of *Frankia* sp. strain BMG5.11 was performed at the Hubbard Center for Genome Studies (University of New Hampshire, Durham, NH) using Illumina technology (16). *Frankia* sp. strain BMG5.11 was grown in defined propionate medium, and high-quality genomic DNA was extracted using the cetyltrimethylammonium bromide (CTAB) method (17). A standard Illumina shotgun library was constructed with a Nextera library preparation kit and sequenced using the Illumina HiSeq 2500 platform, which generated 22,318,488 reads (260-bp insert size) totaling 2,485 Mbp. The Illumina sequence data were trimmed and assembled using CLC Genomics Workbench version 12.0 *de novo* assembly. Default parameters were used for all software unless otherwise specified.

The final draft assembly for *Frankia* sp. strain BMG 5.11 consisted of 219 contigs with a contig *N*_50_ value of 171.5 kb and 214.0× coverage of the genome. The final assembled genome contained a total sequence length of 11,255,272 bp with a G+C content of 69.9%.

The assembled *Frankia* sp. strain BMG5.11 genome was annotated via the NCBI Prokaryotic Genome Annotation Pipeline (PGAP) (18), which resulted in 9,926 candidate protein-encoding genes, 92 tRNAs, 6 noncoding RNAs, and 2 complete rRNA regions. The genome size and corresponding number of coding sequences fit within the values reported for cluster 3 genomes (9). Bioinformatic analysis of this genome using the antiSMASH program (19) revealed the presence of large numbers of secondary metabolic biosynthetic gene clusters, which is consistent with previous results for other *Frankia* lineage 3 genomes, including cluster 3 (9, 20). Many of these potential natural products might be involved in plant-microbe interactions and aid in the plant growth-promoting activities. A whole-genome-based taxonomic analysis via the Type (Strain) Genome Server (TYGS) platform (21) (https://tygs.dsmz.de), including determination of digital DNA-DNA hybridization (dDDH) values (22), was performed to identify this strain in comparison with other *Frankia* genomes. The type-based species clustering using a 70% dDDH radius around each of the type strains was performed as described previously (23), while subspecies clustering was performed using a 79% dDDH threshold, as introduced previously (24).

### Data availability.

This whole-genome shotgun sequence has been deposited in DDBJ/EMBL/GenBank under the accession number SJXI00000000. The version described in this paper is the first version, SJXI01000000. Both the assembly and raw reads are available in DDBJ/ENA/GenBank under BioProject number PRJNA318498 and SRA number SRR8710209, respectively.
